# Effectiveness and acceptability of interventions to improve faecal immunochemical test (FIT) return in both asymptomatic (screening) and symptomatic populations: protocol for a systematic review of qualitative and quantitative evidence

**DOI:** 10.1136/bmjopen-2025-109663

**Published:** 2026-02-16

**Authors:** Naseeb Ezaydi, Matthew Kurien, Oliver Allchin, Katie Biggs, Nupur Chowdhury, David Humes, Ian Kellar, Savita Shanbhag, Jennifer Brown

**Affiliations:** 1 School of Medicine and Population Health, The University of Sheffield, Sheffield, UK; 2 The University Library, The University of Sheffield, Sheffield, UK; 3 Faculty of Medicine and Health Sciences, University of Nottingham, Nottingham, UK; 4 School of Psychology, The University of Sheffield, Sheffield, UK; 5 Hywel Dda University Health Board, Carmarthen, UK

**Keywords:** Early Detection of Cancer, Mass Screening, Gastrointestinal tumours

## Abstract

**Abstract:**

**Introduction:**

Colorectal cancer (CRC) is the fourth most common cancer in the UK and second leading cause of cancer-related deaths. The faecal immunochemical test (FIT) is a non-invasive home-based test used for both symptomatic assessment and population-based screening. However, approximately 30% of screening FIT kits and 10% of symptomatic FIT kits are never returned. Under-served populations, including ethnic minorities, socioeconomically deprived communities and those with mental health conditions, experience particularly low FIT return rates, contributing to health inequalities in CRC outcomes. This systematic review aims to synthesise evidence on the effectiveness and acceptability of interventions to improve FIT returns in both asymptomatic screening and symptomatic populations, with particular focus on under-served communities.

**Methods and analysis:**

We will conduct a systematic review of qualitative and quantitative evidence. We will search Scopus, MedLine via Ovid, CINAHL via Ebsco and Cochrane Central Register of Controlled Trials from September 2010 onwards, supplemented by reference screening and trial registry searches. Eligible studies will include randomised controlled trials, quasi-experimental studies, observational studies, qualitative studies, mixed-methods studies and implementation studies examining FIT interventions in screening or symptomatic populations. Two reviewers will independently screen search results for eligible studies. Data extraction will capture study characteristics, population demographics, intervention components and outcomes including FIT return rates, acceptability, feasibility and implementation factors. Quantitative data will undergo systematic tabulation and meta-analysis where appropriate, with narrative synthesis for heterogeneous studies. Qualitative data will be analysed using framework-based thematic analysis, mapping findings to both the theoretical domains framework and theoretical framework of acceptability. A mixed-methods synthesis will integrate quantitative and qualitative findings to identify intervention characteristics, implementation strategies and contextual factors associated with improved outcomes across different population groups.

**Ethics and dissemination:**

Ethics approval is not required as this systematic review will analyse published studies. Findings will be disseminated through peer-reviewed publication and conference presentations.

**PROSPERO registration number:**

CRD420251111663.

**STRENGTHS AND LIMITATIONS OF THIS STUDY**

- This will be the first systematic review to synthesise both quantitative and qualitative evidence specifically on interventions to improve faecal immunochemical test returns, using a robust mixed-methods approach.
- The review will employ theoretical frameworks (theoretical domains framework and theoretical framework of acceptability) to provide comprehensive understanding of both behavioural mechanisms and acceptability factors.
- The use of automated screening carries a small risk that relevant but poorly indexed studies, particularly qualitative or implementation studies that use non-standard terminology, could be missed by the algorithm.
- The search is limited to papers published from September 2010 onwards, which excludes older behavioural or implementation insights from the era of guaiac faecal occult blood tests that might still be applicable today.

## Rationale

 Colorectal cancer (CRC) is the fourth most common cancer in the UK, and the second leading cause of cancer-related deaths.^1^ An expedited diagnosis and early treatment of CRC improve survival and cure rates, with 95% of patients diagnosed at stage 1 surviving 5 years or more, compared with only 10% of those diagnosed at stage 4.^2^,^4^

The faecal immunochemical test (FIT) is a non-invasive, dipstick test that is completed at an individual’s home and sent for analysis. The test detects the early degradation products of blood in the faeces.^5^,^7^ It is now widely used as a surrogate marker for bowel cancer in two distinct primary care pathways. First, FIT is used for the assessment of symptomatic individuals presenting with potential bowel cancer symptoms, such as changes in bowel habits, abdominal pain, rectal bleeding and weight loss. Second, FIT is used as part of population-based screening programmes for people over the age of 50, regardless of whether or not they are experiencing any symptoms.^5 8^ This is a protocol for a systematic review of evidence about the effectiveness and acceptability of interventions to improve FIT returns in both contexts.

Approximately 30% of CRC screening FIT kits and 10% of symptomatic FIT kits are never returned.^9 10^ This represents about 2.1 million unreturned FIT kits annually, resulting in missed opportunities for early CRC detection and a financial loss of approximately £11.1 million to the NHS,^7^ as well as creating unnecessary non-recyclable waste. To address low FIT returns, programmes and interventions are being developed to identify and overcome barriers to correct FIT completion.

One example of such an intervention is the DETECT-CRC (Early DETECTion of ColoRectal Cancer in Yorkshire) feasibility study, which aims to increase FIT returns in socioeconomically deprived areas through a pharmacy-based active case finding service.^11^ This approach aims to identify individuals who may not recognise their symptoms or who face barriers to accessing healthcare services like primary care by offering FIT kits within community pharmacy settings, which can be completed at home.

A number of systematic reviews have previously examined interventions to improve CRC screening uptake.^12^,^15^ However, these studies have primarily focused on broader population-level interventions or specific screening modalities, without comprehensively examining the evidence specific to underserved groups, or exploring the acceptability and implementation of FIT within these populations. This represents a significant gap in the literature, particularly given the known disparities in CRC outcomes among underserved communities. Understanding both the effectiveness and acceptability of existing interventions is essential for developing strategies that can improve CRC outcomes equitably and efficiently across all population groups.

Comparable with many other cancers,^16^ inequalities are prevalent across the entire CRC pathway: underserved populations, including ethnic minority communities, socioeconomically deprived populations, individuals from certain religious backgrounds, people with serious mental health conditions, those with low educational attainment and transgender individuals, have reduced screening engagement, delayed diagnoses and inequitable access to treatment and follow-up care, which ultimately leads to poorer CRC outcomes.^16^,^21^

### Aims and objectives

The aim of this systematic review will be to synthesise the evidence on the effectiveness and acceptability of interventions to improve FIT returns in both asymptomatic screening and symptomatic populations.

We will review *any* intervention designed to improve FIT return rates in either screening or symptomatic pathways. This intentionally broad scope reflects the heterogeneity of approaches in the literature, which may include (but are not limited to): behavioural interventions (eg, reminders, educational materials, motivational messaging); service delivery modifications (eg, pharmacy-based distribution, community outreach); kit design or distribution method changes (eg, simplified instructions, modified sample collection devices) or multi-component complex interventions combining multiple strategies. One aim of our synthesis will be to develop a typology of interventions from the included studies using the TIDieR (template for intervention description and replication) checklist.^22^

The specific objectives are:

1. To evaluate the effectiveness of interventions to improve FIT completion/return rates and subsequent follow-up in asymptomatic (screening) or symptomatic populations.
2. To assess the acceptability and feasibility of these interventions from the perspective of both service users and providers.
3. To examine how intervention effectiveness varies across different population subgroups, with particular attention to under-served communities (defined below).
4. To examine the implementation of interventions to improve FIT returns, including barriers and facilitators to implementation, implementation strategies used, and contextual factors that influence successful implementation across different healthcare settings and populations.

## Methods

We will undertake a systematic review of qualitative and quantitative evidence about the acceptability and effectiveness of interventions to increase FIT returns. This protocol is reported in line with the preferred reporting items for systematic review and meta-analysis protocols reporting guidelines^23^ (online supplemental file 1) and has been prospectively registered on PROSPERO (CRD420251111663).

### Defining under-served populations

We will specifically evaluate interventions within under-served populations, defined according to the UK government framework as groups experiencing socioeconomic deprivation, any protected characteristics under the UK 2010 Equality Act, barriers to healthcare registration, housing instability, immigration status barriers, institutional barriers, or complex health and social needs.^24^

Within this broader definition, we will specifically target the communities most commonly experiencing bowel cancer inequities, including older adults, people from Black, Asian and minority ethnic backgrounds, those living in areas of high socioeconomic deprivation, men (who have lower screening participation rates), and people with learning disabilities or mental health conditions who face additional barriers to screening access and follow-up care.

Studies will be included if they target these populations specifically, or provide data allowing for assessment of differential intervention effects in different population groups.

### Eligibility criteria

#### Participants/population

Inclusion criteria:

1. Asymptomatic populations: adults (age 18 years or over) who are eligible for CRC screening according to national guidelines.
2. Symptomatic populations: adults (age 18 years or over) presenting with symptoms in primary care that warrant FIT as part of diagnostic evaluation.

Exclusion criteria: non-human studies; studies conducted exclusively in secondary or tertiary care settings; studies focusing on populations with established CRC diagnosis; studies where FIT is used for post-treatment surveillance rather than screening or diagnostic purposes.

In cases where studies report mixed populations, we will include papers provided data for the eligible population of interest can be extracted separately, or, where this is not possible, where the majority of the study sample is eligible according to our criteria.

#### Concept

Inclusion criteria: studies examining the effectiveness and/or acceptability of interventions designed to improve FIT returns. Eligible studies may include: (a) Controlled comparisons (eg, randomised controlled trials (RCTs) or quasi-experimental studies comparing intervention vs usual care/control), (b) Pre-post comparisons (eg, FIT return rates before vs after intervention implementation) or (c) Studies without formal comparators (eg, qualitative studies examining acceptability, implementation studies describing barriers/facilitators). Our primary outcome of “intervention effectiveness (FIT return rates)” will be extracted from controlled or pre-post comparisons where available; however, we will also include studies without quantitative comparators that provide valuable evidence on acceptability, feasibility, and implementation factors.

Exclusion criteria: studies focusing on other CRC detection methods (eg, guaiac faecal occult blood tests, colonoscopy) without specific data on FIT; studies that do not evaluate intervention effectiveness or acceptability (FIT return rates).

#### Context

There will be no geographical restrictions on study inclusion. Studies conducted in any country or healthcare setting will be eligible, regardless of income classification or healthcare system structure. This approach recognises that valuable insights on interventions for under-served populations may emerge from diverse global contexts.

#### Study type

Inclusion criteria: RCTs, quasi-experimental studies, observational studies (cohort, case-control, cross-sectional), qualitative studies, mixed-methods studies and implementation studies. It is anticipated that quantitative study designs (RCTs, quasi-experimental and observational studies) will primarily contribute to effectiveness objectives, while qualitative studies will contribute to implementation and acceptability objectives.

Exclusion criteria: commentary or opinion publications that do not present new data.

Publications in non-English languages will also be excluded.

Searches will be limited to papers published from September 2010 onwards (when FIT started to replace older detection methods).

### Main outcome(s)

Primary outcomes:

- Intervention effectiveness (FIT return rates).

Secondary outcomes:

- Acceptability of interventions designed to improve FIT returns to service users and providers.
- Feasibility of interventions in different settings.
- Barriers and facilitators to implementation.
- Intervention reach across different under-served groups.
- Costs and resource requirements of intervention.

### Information sources

The following bibliographic databases will be searched from 2010 to present: Scopus, MedLine via Ovid, CINAHL via Ebsco and Cochrane Central Register of Controlled Trials. The electronic database search will be supplemented by scanning reference lists of included studies and searching trial registries (ClinicalTrials.gov, WHO International Clinical Trials Registry Platform (ICTRP).

### Search strategy

The search strategy will be designed in consultation with the review team and peer-reviewed by using the peer review of electronic search strategies checklist. See online supplemental file 2 for a sample search strategy.

### Selection of sources of evidence

Study selection will be done in two stages using Rayyan software.^25^ Two reviewers will independently screen titles and abstracts against the eligibility criteria for the first 1000 abstracts. After that, we will employ the Rayyan SVM-based classifier to undertake priority screening. We will auto-eliminate abstracts below 40% likelihood of inclusion, recalculating after every 50 screening decisions made on abstracts that are above the 40% threshold for auto-elimination. The likelihood rating is derived from the distance from the learnt boundary between included and excluded papers within the training set. Abstracts that meet the criteria or provide insufficient information to determine eligibility will proceed to full-text review. Two reviewers will then independently assess the full-text articles for final inclusion. Any disagreements at either stage will be resolved through discussion or consultation with a third reviewer if needed. The reasons for exclusion at the full-text stage will be documented.

### Data extraction process

A standardised data extraction form will be developed and piloted on a sample of included studies. Two reviewers will independently extract data from each study, with discrepancies resolved through discussion or a third reviewer. The form will be iteratively updated to ensure all relevant data are captured. If critical information is missing from any studies, the authors will be contacted by email with a follow-up reminder after 2 weeks if no response is received.

Quantitative results will be extracted for any direct measures of intervention effectiveness relating to FIT uptake and/or return rates.

For qualitative data, we will extract interpretative text and overarching themes as reported by study authors. The extraction of qualitative findings will be structured according to the theoretical framework of acceptability (TFA)^26^ to support subsequent framework analysis. Data will be organised within the TFA’s seven domains: affective attitude, burden, ethicality, intervention coherence, opportunity costs, perceived effectiveness and self-efficacy. Additionally, we will categorise the acceptability outcomes reported in each study as prospective (anticipated acceptability), concurrent (experienced acceptability during intervention delivery) or retrospective (acceptability reflected on after intervention completion).

### Data items

All studies that meet the inclusion criteria will be described in terms of:

- Study characteristics (design, country, setting, sample size, duration).
- Population characteristics (age, sex, ethnicity, socioeconomic status).
- Intervention characteristics (screening or symptomatic pathway, intervention components, implementation strategies) using the TIDieR checklist.^22^
- Theoretical frameworks used in intervention design or evaluation.
- Effectiveness outcomes (FIT uptake, return rates).
- Contextual factors influencing implementation.
- Barriers and facilitators to implementation.
- Adaptations made for specific underserved groups.

### Quality assessment

The mixed methods appraisal tool will be used for qualitative, quantitative and mixed methods studies.

Two reviewers will independently assess quality, with disagreements resolved through consensus or a third reviewer. Quality assessment results will be presented in tables and considered in the interpretation of findings. All eligible studies will be included in the synthesis regardless of quality, but sensitivity analyses may be conducted to explore the impact of quality on results.

### Synthesis of results

Quantitative data will be synthesised through systematic tabulation and, where appropriate, meta-analysis. Where meta-analysis is not appropriate due to heterogeneity in study designs, populations or interventions, we will conduct a narrative synthesis. Meta-analyses will calculate risk ratios or ORs with 95% CIs for dichotomous outcomes (eg, FIT return rates) and mean differences with SD for continuous outcomes (eg, time to return).

Qualitative data will undergo framework-based thematic analysis. Data will be deductively mapped to the theoretical domains framework to identify behavioural determinants influencing FIT uptake and return.^27^ Concurrently, qualitative findings will be cross-mapped to the TFA to examine intervention acceptability.^26^ This dual mapping will allow us to understand both the behavioural mechanisms underlying intervention effects and the acceptability factors that influence implementation success.

We will conduct a mixed-methods synthesis to harness the strengths of both quantitative and qualitative evidence. We will use a convergent synthesis strategy. In a convergent approach, we will simultaneously analyse quantitative and qualitative data independently before merging the findings to create an integrated understanding. This mixed-methods approach is designed to produce a more comprehensive and nuanced understanding than would be possible through isolated examination of either quantitative or qualitative evidence alone.

The strength of evidence will be assessed using Grading of Recommendations, Assessment, Development and Evaluation (GRADE) for quantitative outcomes^28^ and CERQual for qualitative findings.^29^

#### Integration of findings

The findings from both syntheses will be integrated by linking intervention characteristics, implementation strategies and effectiveness outcomes. This integration will help identify which implementation approaches are associated with better outcomes in which contexts and for which populations.

If the data permits, we will produce:

- A meta-analysis of effectiveness and implementation findings.
- A typology of interventions designed to increase FIT returns.
- A summary of implementation strategies associated with intervention success.
- Context-mechanism-outcome configurations to explain what works, for whom, in what circumstances.
- Recommendations for implementing equitable FIT interventions.

The findings from this systematic review will directly inform the development of the intervention package and implementation plan in the PRO-FIT project, ensuring that it builds on existing evidence of what works in promoting FIT use among underserved communities.

### Patient and public involvement

A patient and public involvement panel for the wider PRO-FIT project will review the findings of the systematic review.

## Discussion

A mixed-methods systematic review incorporating qualitative and quantitative evidence is warranted to comprehensively assess the effectiveness, acceptability and implementation of FIT in underserved groups. This approach will allow for a holistic understanding of the barriers and facilitators to FIT uptake and return, as well as the identification of strategies to improve participation and reduce inequities. The integration of qualitative and quantitative evidence will provide a more nuanced understanding of the complex factors influencing FIT use in these populations, which is essential to inform the development of targeted interventions.

Our systematic review will make several important contributions to the existing evidence base. First, it will provide the first comprehensive synthesis of interventions specifically designed to improve FIT returns in underserved populations, addressing a significant gap in the current literature. Unlike previous reviews that have focused on broader population-level interventions or specific screening modalities, this review will specifically examine how interventions perform across different underserved groups, providing crucial insights into health equity considerations. Second, by employing a mixed-methods approach, the review will generate evidence on both what works (effectiveness) and why it works (acceptability and feasibility), providing relevant insights for intervention development. This will also enable the identification of intervention components that are both effective and implementable in real-world settings. Thirdly, the review will produce a typology of interventions that could be used by healthcare providers, policymakers and researchers to design more effective and equitable FIT interventions.

There are important implications for clinical practice and healthcare policy by undertaking this review. As evidence is being collected across diverse contexts, the review will help healthcare providers and policymakers understand what interventions are most effective in different underserved populations. This evidence could inform guidelines on FIT implementation. In addition, our review will identify which approaches are most successful for addressing specific barriers, which are faced by different underserved populations.

Additionally, the review findings will directly inform the development of an intervention package and implementation plan within the National Institute for Health Research funded PRO-FIT project. This ensures a clear pathway from evidence synthesis to intervention development and implementation.

In conclusion, this systematic review will provide crucial evidence to inform the development of more effective and equitable FIT interventions for underserved populations. By understanding both what works and why it works, the review will support the translation of research evidence into practice and policy, ultimately contributing to the reduction of CRC inequalities.

## Supplementary material

10.1136/bmjopen-2025-109663 online supplemental file 1

10.1136/bmjopen-2025-109663 online supplemental file 2

## References

[1] Cancer Research UK (2025). Bowel cancer statistics. https://www.cancerresearchuk.org/health-professional/cancer-statistics/statistics-by-cancer-type/bowel-cancer.

[2] Hamilton W, Walter FM, Rubin G (2016). Improving early diagnosis of symptomatic cancer. Nat Rev Clin Oncol.

[3] Cancer Research UK Survival Bowel cancer.

[4] Neal RD, Tharmanathan P, France B (2015). Is increased time to diagnosis and treatment in symptomatic cancer associated with poorer outcomes? Systematic review. Br J Cancer.

[5] Monahan KJ, Davies MM, Abulafi M (2022). Faecal immunochemical testing (FIT) in patients with signs or symptoms of suspected colorectal cancer (CRC): a joint guideline from the Association of Coloproctology of Great Britain and Ireland (ACPGBI) and the British Society of Gastroenterology (BSG). Gut.

[6] Tinmouth J, Lansdorp-Vogelaar I, Allison JE (2015). Faecal immunochemical tests versus guaiac faecal occult blood tests: what clinicians and colorectal cancer screening programme organisers need to know. Gut.

[7] NICE Quantitative faecal immunochemical tests to guide referral for colorectal cancer in primary care | guidance. https://www.nice.org.uk/guidance/htg690/chapter/2-The-diagnostic-tests.

[8] NICE Overview | quantitative faecal immunochemical testing to guide colorectal cancer pathway referral in primary care | guidance. https://www.nice.org.uk/guidance/htg690.

[9] Robb KA, Kotzur M, Young B (2023). Increasing uptake of FIT colorectal screening: protocol for the TEMPO randomised controlled trial testing a suggested deadline and a planning tool. BMJ Open.

[10] Bailey JA, Morton AJ, Jones J (2023). Sociodemographic variations in the uptake of faecal immunochemical tests in primary care: a retrospective study. Br J Gen Pract.

[11] ISRCTN ISRCTN14156362: Early DETECTion of ColoRectal Cancer in Yorkshire (DETECT-CRC): Feasibility study assessing active case finding of colorectal cancer in socio-economically deprived areas.

[12] Dougherty MK, Brenner AT, Crockett SD (2018). Evaluation of Interventions Intended to Increase Colorectal Cancer Screening Rates in the United States: A Systematic Review and Meta-analysis. JAMA Intern Med.

[13] Issaka RB, Avila P, Whitaker E (2019). Population health interventions to improve colorectal cancer screening by fecal immunochemical tests: A systematic review. Prev Med.

[14] Myers L, Goodwin B, Ralph N (2020). Implementation Strategies for Interventions Aiming to Increase Participation in Mail-Out Bowel Cancer Screening Programs: A Realist Review. *Front Oncol*.

[15] Goodwin BC, Ireland MJ, March S (2019). Strategies for increasing participation in mail-out colorectal cancer screening programs: a systematic review and meta-analysis. Syst Rev.

[16] Mihor A, Tomsic S, Zagar T (2020). Socioeconomic inequalities in cancer incidence in Europe: a comprehensive review of population-based epidemiological studies. Radiol Oncol.

[17] Finke I, Seppä K, Malila N (2021). Educational inequalities and regional variation in colorectal cancer survival in Finland. Cancer Epidemiol.

[18] Percac-Lima S, Ashburner JM, Zai AH (2016). Patient Navigation for Comprehensive Cancer Screening in High-Risk Patients Using a Population-Based Health Information Technology System: A Randomized Clinical Trial. JAMA Intern Med.

[19] Solmi M, Firth J, Miola A (2020). Disparities in cancer screening in people with mental illness across the world versus the general population: prevalence and comparative meta-analysis including 4 717 839 people. Lancet Psychiatry.

[20] Campbell C, Douglas A, Williams L (2020). Are there ethnic and religious variations in uptake of bowel cancer screening? A retrospective cohort study among 1.7 million people in Scotland. BMJ Open.

[21] Stoffel ST, McGregor L, Hirst Y (2022). Evaluation of the Call for a Kit intervention to increase bowel cancer screening uptake in Lancashire, England. J Med Screen.

[22] Hoffmann TC, Glasziou PP, Boutron I (2014). Better reporting of interventions: template for intervention description and replication (TIDieR) checklist and guide. BMJ.

[23] Shamseer L, Moher D, Clarke M (2015). Preferred reporting items for systematic review and meta-analysis protocols (PRISMA-P) 2015: elaboration and explanation. BMJ.

[24] Population screening: review of interventions to improve participation among underserved groups - GOV.UK.

[25] Ouzzani M, Hammady H, Fedorowicz Z (2016). Rayyan-a web and mobile app for systematic reviews. Syst Rev.

[26] Sekhon M, Cartwright M, Francis JJ (2017). Acceptability of healthcare interventions: an overview of reviews and development of a theoretical framework. BMC Health Serv Res.

[27] Cane J, O’Connor D, Michie S (2012). Validation of the theoretical domains framework for use in behaviour change and implementation research. Implementation Sci.

[28] Guyatt G, Oxman AD, Akl EA (2011). GRADE guidelines: 1. Introduction—GRADE evidence profiles and summary of findings tables. J Clin Epidemiol.

[29] Lewin S, Booth A, Glenton C (2018). Applying GRADE-CERQual to qualitative evidence synthesis findings: introduction to the series. Implementation Sci.

