# Supplementary material for: Effectiveness and acceptability of interventions to improve faecal immunochemical test (FIT) return in both asymptomatic (screening) and symptomatic populations: protocol for a systematic review of qualitative and quantitative evidence
Source: BMJ Open. 2026 Feb 16;16(2):e109663. doi: 10.1136/bmjopen-2025-109663 (PMC12911743; doi:10.1136/bmjopen-2025-109663)
Supplement: online supplemental file 2 [file bmjopen-16-2-s002.docx]

**Supplemental File 2:**

**Search strategies:**

## Scopus

"Faecal immunochemical test*" OR "fecal immunochemical test*" OR FIT OR "fecal occult blood test*" OR "faecal occult blood test*" OR FOBT OR "stool test*" OR "screening kit" OR "home test*" OR ((feces OR faeces OR stool OR fecal OR faecal OR FOBT) W/3 (immunoassay OR immunochemi* OR immunohistochemi*))

AND

CRC OR “colorectal cancer” OR “bowel cancer” OR “colon* cancer” OR “rectal cancer” OR “colorectal neoplasm*”

AND

adhere* OR comply OR complian* OR complie* OR complying OR cooperat* OR co-operat* OR accept* OR non-complian* OR noncomplian* OR non-adheren* OR nonadheren* OR adhere* OR return* OR uptake OR complet* OR participat* OR non-participat* OR nonparticipat* OR non-attendance OR “non attendance” OR barrier* OR withdraw* OR refuse OR refusal* OR refusing OR use OR likelihood OR attitude* OR perception* OR perceive* OR belief* OR believ* OR behavior* OR facilitator* OR access* OR anxiet* OR aware* OR motivat* OR view* OR rural OR underserved OR dropout* OR drop-out* OR "drop* out*"

AND

intervention* OR program* OR strateg* OR educat* OR inform* OR remind* OR prompt* OR text OR SMS OR phone OR telephone OR outreach* OR incentiv* OR reimburse* OR payment* OR “health fair*” OR "shared medical appointment" OR "one on one" OR “one to one” OR “1 to 1” OR "1 on 1" OR encourag* OR deadline* OR personalis* OR personaliz* OR invit* OR communicat* OR support* OR "face to face" OR face-to-face OR in-person OR "in person" OR advert* OR endorse* OR signpost* OR “health literacy” OR discuss* OR conversation* OR leaflet* OR instruction* OR guidance OR guide* OR training OR mediat* OR target* OR pharmac* OR communit* OR partner* OR interview* OR mail* OR letter OR post OR “social media” OR GP OR “general practitioner*” OR follow-up OR “follow up” OR tool* OR plan OR planning OR service OR provid* OR outreach OR "cultural* tailor*" OR "cultural* sensitiv*" OR navigat* OR video*

**(search run 17/09/2025) 3,253 results in Scopus when limited to 2010 onwards.**

## CINAHL via EBSCO

"Faecal immunochemical test*" OR "fecal immunochemical test*" OR FIT OR "fecal occult blood test*" OR "faecal occult blood test*" OR FOBT OR "stool test*" OR "screening kit" OR "home test*" OR ((feces OR faeces OR stool OR fecal OR faecal OR FOBT) N3 (immunoassay OR immunochemi* OR immunohistochemi*))

AND

CRC OR “colorectal cancer” OR “bowel cancer” OR “colon* cancer” OR “rectal cancer” OR “colorectal neoplasm*”

AND

adhere* OR comply OR complian* OR complie* OR complying OR cooperat* OR co-operat* OR accept* OR non-complian* OR noncomplian* OR non-adheren* OR nonadheren* OR adhere* OR return* OR uptake OR complet* OR participat* OR non-participat* OR nonparticipat* OR non-attendance OR “non attendance” OR barrier* OR withdraw* OR refuse OR refusal* OR refusing OR use OR likelihood OR attitude* OR perception* OR perceive* OR belief* OR believ* OR behavior* OR facilitator* OR access* OR anxiet* OR aware* OR motivat* OR view* OR rural OR underserved OR dropout* OR drop-out* OR "drop* out*"

AND

intervention* OR program* OR strateg* OR educat* OR inform* OR remind* OR prompt* OR text OR SMS OR phone OR telephone OR outreach* OR incentiv* OR reimburse* OR payment* OR “health fair*” OR "shared medical appointment" OR "one on one" OR “one to one” OR “1 to 1” OR "1 on 1" OR encourag* OR deadline* OR personalis* OR personaliz* OR invit* OR communicat* OR support* OR "face to face" OR face-to-face OR in-person OR "in person" OR advert* OR endorse* OR signpost* OR “health literacy” OR discuss* OR conversation* OR leaflet* OR instruction* OR guidance OR guide* OR training OR mediat* OR target* OR pharmac* OR communit* OR partner* OR interview* OR mail* OR letter OR post OR “social media” OR GP OR “general practitioner*” OR follow-up OR “follow up” OR tool* OR plan OR planning OR service OR provid* OR outreach OR "cultural* tailor*" OR "cultural* sensitiv*" OR navigat* OR video*

**All fields, Search options: Find all my search terms, no expanders**

**(search run 17/09/2025) 1,207 results when limited to 01/01/2010 to 31/12/2025**

## CENTRAL ([Cochrane Central Register of Controlled Trials](https://www.cochranelibrary.com/en/central/about-central))

1349 Trials matching "Faecal immunochemical test" OR "fecal immunochemical test" OR FIT OR "fecal occult blood test" OR "faecal occult blood test" OR FOBT OR "stool test" OR "screening kit" OR "home test" OR ((feces OR faeces OR stool OR fecal OR faecal OR FOBT) NEAR/3 (immunoassay OR immunochemi* OR immunohistochemi*)) in Title Abstract Keyword AND CRC OR “colorectal cancer” OR “bowel cancer” OR (colon* NEXT cancer) OR “rectal cancer” OR (colorectal NEXT neoplasm*) in Title Abstract Keyword AND adhere* OR comply OR complian* OR complie* OR complying OR cooperat* OR co-operat* OR accept* OR non-complian* OR noncomplian* OR non-adheren* OR nonadheren* OR adhere* OR return* OR uptake OR complet* OR participat* OR non-participat* OR nonparticipat* OR non-attendance OR “non attendance” OR barrier* OR withdraw* OR refuse OR refusal* OR refusing OR use OR likelihood OR attitude* OR perception* OR perceive* OR belief* OR believ* OR behavior* OR facilitator* OR access* OR anxiet* OR aware* OR motivat* OR view* OR rural OR underserved OR dropout* OR drop-out* OR (drop* NEXT out*) in Title Abstract Keyword AND intervention* OR program* OR strateg* OR educat* OR inform* OR remind* OR prompt* OR text OR SMS OR phone OR telephone OR outreach* OR incentiv* OR reimburse* OR payment* OR (health NEXT fair*) OR "shared medical appointment" OR "one on one" OR “one to one” OR “1 to 1” OR "1 on 1" OR encourag* OR deadline* OR personalis* OR personaliz* OR invit* OR communicat* OR support* OR "face to face" OR face-to-face OR in-person OR "in person" OR advert* OR endorse* OR signpost* OR “health literacy” OR discuss* OR conversation* OR leaflet* OR instruction* OR guidance OR guide* OR training OR mediat* OR target* OR pharmac* OR communit* OR partner* OR interview* OR mail* OR letter OR post OR “social media” OR GP OR (general NEXT practitioner*) OR follow-up OR “follow up” OR tool* OR plan OR planning OR service OR provid* OR outreach OR (cultural* NEXT tailor*) OR (cultural* NEXT sensitiv*) OR navigat* OR video* in Title Abstract Keyword - with Publication Year from 2010 to 2025, with Cochrane Library publication date Between Jan 2010 and Dec 2025, in Trials (Word variations have been searched)

**(search run 17/09/2025) 1349 results when limited to January 2010 onwards.**

## Medline via OVID

Ovid MEDLINE(R) Epub Ahead of Print and In-Process, In-Data-Review & Other Non-Indexed Citations and Daily <September 16, 2025>

1 exp Occult Blood/ 6781

2 Feces/ 110599

3 exp Immunoassay/ 507591

4 Immunochemistry/ 9663

5 Immunohistochemistry/ 307772

6 3 or 4 or 5 779659

7 2 and 6 5466

8 1 or 7 12022

9 ("Faecal immunochemical test*" or "fecal immunochemical test*" or FIT or "fecal occult blood test*" or "faecal occult blood test*" or FOBT or "stool test*" or "screening kit" or "home test*" or ((feces or faeces or stool or fecal or faecal or FOBT) adj3 (immunoassay or immunochemi* or immunohistochemi*))).mp. [mp=title, book title, abstract, original title, name of substance word, subject heading word, floating sub-heading word, keyword heading word, organism supplementary concept word, protocol supplementary concept word, rare disease supplementary concept word, unique identifier, synonyms, population supplementary concept word, anatomy supplementary concept word] 190329

10 8 or 9 198355

11 exp Colorectal Neoplasms/ 259199

12 (CRC or "colorectal cancer" or "bowel cancer" or "colon* cancer" or "rectal cancer").mp. [mp=title, book title, abstract, original title, name of substance word, subject heading word, floating sub-heading word, keyword heading word, organism supplementary concept word, protocol supplementary concept word, rare disease supplementary concept word, unique identifier, synonyms, population supplementary concept word, anatomy supplementary concept word] 237084

13 11 or 12 335801

14 exp "Patient Acceptance of Health Care"/ 248636

15 exp Patient Compliance/ 154938

16 exp "Treatment Adherence and Compliance"/ 360194

17 No-Show Patients/ 319

18 Patient Dropouts/ 8578

19 (adhere* or comply or complian* or complie* or complying or cooperat* or co-operat* or accept* or non-complian* or noncomplian* or non-adheren* or nonadheren* or adhere* or return* or uptake or complet* or participat* or non-participat* or nonparticipat* or non-attendance or "non attendance" or barrier* or withdraw* or refuse or refusal* or refusing or "use" or likelihood or attitude* or perception* or perceive* or belief* or believ* or behavior* or facilitator* or access* or anxiet* or aware* or motivat* or view* or rural or underserved).mp. [mp=title, book title, abstract, original title, name of substance word, subject heading word, floating sub-heading word, keyword heading word, organism supplementary concept word, protocol supplementary concept word, rare disease supplementary concept word, unique identifier, synonyms, population supplementary concept word, anatomy supplementary concept word] 13396046

20 14 or 15 or 16 or 17 or 18 or 19 13455285

21 exp Adherence Interventions/ 65364

22 exp Reminder Systems/ 4009

23 exp Text Messaging/ 5233

24 exp Health Education/ 275342

25 exp Health Promotion/ 90462

26 exp Patient Education as Topic/ 91564

27 exp Patient Education Handout/ 6033

28 exp Consumer Health Information/ 16588

29 exp Health Fairs/ 492

30 exp Plain Language Summaries/ 32

31 exp Health Communication/ 3715

32 exp Health Literacy/ 12316

33 exp Social Media/ 20556

34 exp Internet-Based Intervention/ 1832

35 exp Interactive Tutorial/ 276

36 exp Webcast/ 1154

37 (intervention* or program* or strateg* or educat* or inform* or remind* or prompt* or text or SMS or phone or telephone or outreach* or incentiv* or reimburse* or payment* or "health fair*" or "shared medical appointment" or "one on one" or "one to one" or "1 to 1" or "1 on 1" or encourag* or deadline* or personalis* or personaliz* or invit* or communicat* or support* or "face to face" or face-to-face or in-person or "in person" or advert* or endorse* or signpost* or "health literacy" or discuss* or conversation* or leaflet* or instruction* or guidance or guide* or training or mediat* or target* or pharmac* or communit* or partner* or interview* or mail* or letter or post or "social media" or GP or "general practitioner*" or follow-up or "follow up*" or tool* or plan or planning or service or provid* or outreach or "cultural* tailor*" or "cultural* sensitiv*" or navigat* or video*).mp. [mp=title, book title, abstract, original title, name of substance word, subject heading word, floating sub-heading word, keyword heading word, organism supplementary concept word, protocol supplementary concept word, rare disease supplementary concept word, unique identifier, synonyms, population supplementary concept word, anatomy supplementary concept word] 25261498

38 21 or 22 or 23 or 24 or 25 or 26 or 27 or 28 or 29 or 30 or 31 or 32 or 33 or 34 or 35 or 36 or 37 25277171

39 10 and 13 and 20 and 38 4925

40 limit 39 to yr="2010 -Current" 3526

**search run 17/09/2025) 3526 results when limited to 2010 - current**
