# Supplementary material for: Effectiveness and acceptability of interventions to improve faecal immunochemical test (FIT) return in both asymptomatic (screening) and symptomatic populations: protocol for a systematic review of qualitative and quantitative evidence
Source: BMJ Open. 2026 Feb 16;16(2):e109663. doi: 10.1136/bmjopen-2025-109663 (PMC12911743; doi:10.1136/bmjopen-2025-109663)
Supplement: Supplementary file 3 [file bmjopen-2025-109663.reviewer_comments.pdf]

PEER REVIEW HISTORY

BMJ Open publishes all reviews undertaken for accepted manuscripts. Reviewers are asked to complete a checklist review form and are provided with free text boxes to elaborate on their assessment. These free text comments are reproduced below.

ARTICLE DETAILS

Title (Provisional)

Effectiveness and acceptability of interventions to improve Faecal Immunochemical Test (FIT) return in both asymptomatic (screening) and symptomatic populations: protocol for a systematic review of qualitative and quantitative evidence

Authors

Ezaydi, Naseeb; Kurien, Matthew; Allchin, Oliver; Biggs, Katie; Chowdhury, Nupur; Humes, David; Kellar, Ian; Shanbhag, Savita; Brown, Jennifer

---

VERSION 1 - REVIEW

**Reviewer**	**1**
**Name**	**Farr, Deeonna**
**Affiliation**	**East Carolina University**
**Date**	**01-Nov-2025**
**COI**	**None**

The authors present a protocol for systematic review of interventions to increase the FIT test return rates for: 1) population level screening and 2) diagnostic testing among underserved populations. The review has the potential to make important contributions to this topic, however several aspects of the review have not been adequately conceptualized and/or require additional details. My detailed comments are presented below:

Introduction

• Overall rationale make sense however there is existing research that addresses some of the proposed research question:

Belon AP, McKenzie E, Teare G, et al. Effective strategies for Fecal Immunochemical Tests (FIT) programs to improve colorectal cancer screening uptake among populations with limited access to the healthcare system: a rapid review. BMC Health Serv Res. 2024;24(1):128. doi:10.1186/s12913-024-10573-4

• The third paragraph discusses FIT test performance which is not relevant to the research question nor can be assessed with the proposed study design

• Objectives are not fully described as the type of interventions reviewed (the strategies being implemented to improve FIT return rates) are never outlined and comparators are not defined (improve return rates implies a comparison to another group/time point etc)

Methods

• The definition of underserved communities is relative to country context as the designation is a result historical and social factors. The UK definition of underserved populations only applies to the UK and can not applied to other countries across the globe. If the authors plan to use the UK designation, then studies outside of the UK must be excluded. It is only appropriate to evaluation studies across countries if the category is the same across context (e.g. age, biological sex, low income, rural, immigration/citizenship status etc)

• Examination of cancer detection rates is outside the scope of the proposed review will consist of studies of intervention to improve return rates and not epidemiological studies of FIT performance.

• The description of the abstract review process is not clear. How is a 40% probability of inclusion defined?

• The extraction of qualitative data should be limited to themes only as quotes are individual data points which would be comparable to pulling the test return time frame of one participant in an intervention study

• Intervention characteristics in the data item section are not described in appropriate detail

• As this is a protocol paper, the synthesis strategy for examining quantitative and qualitative results needs to be defined in advance.

• The authors propose meta-analyses but do not describe quantitative analysis methods

• One of proposed outputs is "a narrative synthesis on which population subgroups are underserved by FIT interventions" This does not align with the stated goal of examine the effects of interventions/intervention strategies on return rates in specific populations. What does "underserved by FIT interventions" mean?

• No discussion of evaluating the strength of evidence or meta-biases is offered.

VERSION 1 - AUTHOR RESPONSE

Reviewer: 1
Dr. Deeonna Farr, East Carolina University
Comments to the Author:
The authors present a protocol for systematic review of interventions to increase the FIT test return rates for: 1) population level screening and 2) diagnostic testing among underserved populations. The review has the potential to make important contributions

to this topic, however several aspects of the review have not been adequately conceptualized and/or require additional details. My detailed comments are presented below:

Introduction

• Overall rationale make sense however there is existing research that addresses some of the proposed research question:
Belon AP, McKenzie E, Teare G, et al. Effective strategies for Fecal Immunochemical Tests (FIT) programs to improve colorectal cancer screening uptake among populations with limited access to the healthcare system: a rapid review. BMC Health Serv Res. 2024;24(1):128. doi:10.1186/s12913-024-10573-4

We thank the reviewer for highlighting the Belon et al. (2024) systematic review. We are familiar with this work and believe that our proposed review offers important complementary contributions to the literature. Although Belon et al. examined strategies specifically in screening contexts, our review encompasses both asymptomatic (screening) and symptomatic populations. Whilst both reviews address populations with limited healthcare access, our review will specifically use the comprehensive UK government framework definition of underserved populations. Our review will also integrate quantitative effectiveness data with qualitative evidence on acceptability, feasibility, and implementation using established theoretical frameworks, which is different to the Belon et al. work. We believe that this will help provide insights for future intervention design and implementation. Importantly, we will ensure appropriate citation and discussion of Belon et al.'s findings occur in our completed manuscript, which would help contextualise our work within the existing evidence base.

• The third paragraph discusses FIT test performance which is not relevant to the research question nor can be assessed with the proposed study design

Paragraph deleted.

• Objectives are not fully described as the type of interventions reviewed (the strategies being implemented to improve FIT return rates) are never outlined and comparators are not defined (improve return rates implies a comparison to another group/time point etc)

We thank the reviewer for this helpful feedback. We agree that further clarity is needed regarding our intervention types and comparators. This is now adjusted in the objectives (page 4) to read:

We will review any intervention designed to improve FIT completion and/ or return rates in either screening or symptomatic pathways. This intentionally broad scope reflects the heterogeneity of approaches in the literature, which may include (but are not limited to): behavioural interventions (e.g., reminders, educational materials, motivational messaging); service delivery modifications (e.g., pharmacy-based distribution, community outreach); kit design or distribution method changes; or multi-component complex interventions combining multiple strategies. One aim of our synthesis will be to develop a typology of interventions from the included studies using the TIDieR (Template for Intervention Description and Replication) checklist.

We have also provided further text in our eligibility criteria (Methods section, page 5) to read:

Inclusion criteria - Studies examining the effectiveness and/or acceptability of FIT interventions. Eligible studies may include: (a) Controlled comparisons (e.g., RCTs or quasi-experimental studies comparing intervention vs. usual care/control); (b) Pre-post comparisons (e.g., FIT return rates before vs. after intervention implementation); or (c) studies without formal comparators (e.g., qualitative studies examining acceptability, implementation studies describing barriers/facilitators). Our primary outcome of "intervention effectiveness (FIT return rates)" will be extracted from controlled or pre-post comparisons where available; however, we will also include studies without quantitative comparators that provide valuable evidence on acceptability, feasibility, and implementation factors.

Methods

• The definition of underserved communities is relative to country context as the designation is a result historical and social factors. The UK definition of underserved populations only applies to the UK and can not applied to other countries across the globe. If the authors plan to use the UK designation, then studies outside of the UK must be excluded. It is only appropriate to evaluation studies across countries if the category is the same across context (e.g. age, biological sex, low income, rural, immigration/citizenship status etc)

We thank the reviewer for raising this important consideration about the context-specific nature of underserved population definitions. We respectfully would wish to maintain our current approach of including non-UK studies for the following reasons:

- The UK government framework definition serves as a broad conceptual lens to identify those populations experiencing health inequalities. It encompasses socioeconomic deprivation, protected characteristics, healthcare access barriers, and complex needs. These are universal dimensions of health inequality that manifest across global contexts, even if the specific operationalisations differ by country.
- Our extraction approach captures how each study defines its target population within its own context. We are not imposing UK-specific thresholds or classifications onto international studies. Rather, we will recognise that a study

targeting "socioeconomically deprived" populations in the US, "low-income" groups in Canada, or "Index of Multiple Deprivation quintile 5" in the UK are all addressing similar underlying health inequalities, even if measured differently.

- Given the known disparities in CRC outcomes among underserved populations globally, restricting our review to UK-only studies would severely limit the evidence base for an already under-researched area, and potentially exclude effective interventions developed in other healthcare systems.
-

• Examination of cancer detection rates is outside the scope of the proposed review will consist of studies of intervention to improve return rates and not epidemiological studies of FIT performance.

We agree with the reviewer and have removed this from our secondary objectives (page 7)

• The description of the abstract review process is not clear. How is a 40% probability of inclusion defined?

We have now clarified this in the section below.

Two reviewers will independently screen titles and abstracts against the eligibility criteria for the first 1000 abstracts . After that, we will employ the Rayyan SVM based classifier to undertake priority screening. We will auto-eliminate abstracts below 40% likelihood of inclusion, recalculating after every 50 screening decisions made on abstracts that are above the 40% threshold for auto-elimination. The likelihood rating is derived from the distance from the learned boundary between included and excluded papers within the training set.

• The extraction of qualitative data should be limited to themes only as quotes are individual data points which would be comparable to pulling the test return time frame of one participant in an intervention study

We thank the reviewer for this important methodological consideration. We agree that extracting individual participant quotes could introduce unnecessary granularity. We have revised our data extraction approach to focus on interpretative text and overarching themes as reported by study authors, rather than extracting individual participant quotes.

The revised text in the Data Extraction Process section now reads:

"For qualitative data, we will extract interpretative text, and overarching themes as reported by study authors. The extraction of qualitative findings will be structured according to the Theoretical Framework of Acceptability (TFA) (25) to support subsequent framework analysis

- Intervention characteristics in the data item section are not described in appropriate detail

Thanks to the reviewer for highlighting this. This has now been addressed in response to the earlier by detailing the intervention characteristics (page 4).

- As this is a protocol paper, the synthesis strategy for examining quantitative and qualitative results needs to be defined in advance.

The following synthesis strategy has been defined:

Quantitative data will be synthesised through systematic tabulation and, where appropriate, meta-analysis. Where meta-analysis is not appropriate due to heterogeneity in study designs, populations, or interventions, we will conduct a narrative synthesis.

Qualitative data will undergo framework-based thematic analysis. Data will be deductively mapped to the Theoretical Domains Framework (TDF) to identify behavioural determinants influencing FIT uptake and return (27). Concurrently, qualitative findings will be cross-mapped to the Theoretical Framework of Acceptability (TFA) to examine intervention acceptability (25). This dual mapping will allow us to understand both the behavioural mechanisms underlying intervention effects and the acceptability factors that influence implementation success.

We will conduct a mixed-methods synthesis to harness the strengths of both quantitative and qualitative evidence. We will utilise a convergent synthesis strategy. In a convergent approach, we will simultaneously analyse quantitative and qualitative data independently before merging the findings to create an integrated understanding. This mixed-methods approach is designed to produce a more comprehensive and nuanced understanding than would be possible through isolated examination of either quantitative or qualitative evidence alone.

- The authors propose meta-analyses but do not describe quantitative analysis methods

The following has been added:

“Meta-analyses will calculate risk ratios or odds ratios with 95% confidence intervals for dichotomous outcomes (e.g., FIT return rates) and mean differences with standard deviations for continuous outcomes (e.g., time to return). “

• One of proposed outputs is “a narrative synthesis on which population subgroups are underserved by FIT interventions” This does not align with the stated goal of examine the effects of interventions/intervention strategies on return rates in specific populations. What does “underserved by FIT interventions” mean?

Output has been removed.

• No discussion of evaluating the strength of evidence or meta-biases is offered.

The following has been added: The strength of evidence will be assessed using GRADE for quantitative outcomes (28) and CERQual for qualitative findings (29).
